# Production of succinate by engineered strains of *Synechocystis* PCC 6803 overexpressing phosphoenolpyruvate carboxylase and a glyoxylate shunt

**DOI:** 10.1186/s12934-021-01529-y

**Published:** 2021-02-08

**Authors:** Claudia Durall, Kateryna Kukil, Jeffrey A. Hawkes, Alessia Albergati, Peter Lindblad, Pia Lindberg

**Affiliations:** 1 Microbial Chemistry, Department of Chemistry-Ångström, Uppsala University, Box 523, 751 20 Uppsala, Sweden; 2 Analytical Chemistry, Department of Chemistry-BMC, Uppsala University, Box 599, 751 20 Uppsala, Sweden

**Keywords:** *Synechocystis* PCC 6803, TCA cycle, Glyoxylate shunt, Succinate, Phosphoenolpyruvate carboxylase, Succinate dehydrogenase, Acetate, 2-thenoyltrifluoroacetone

## Abstract

**Background:**

Cyanobacteria are promising hosts for the production of various industrially important compounds such as succinate. This study focuses on introduction of the glyoxylate shunt, which is naturally present in only a few cyanobacteria, into *Synechocystis* PCC 6803. In order to test its impact on cell metabolism, engineered strains were evaluated for succinate accumulation under conditions of light, darkness and anoxic darkness. Each condition was complemented by treatments with 2-thenoyltrifluoroacetone, an inhibitor of succinate dehydrogenase enzyme, and acetate, both in nitrogen replete and deplete medium.

**Results:**

We were able to introduce genes encoding the glyoxylate shunt, *aceA* and *aceB,* encoding isocitrate lyase and malate synthase respectively, into a strain of *Synechocystis* PCC 6803 engineered to overexpress phosphoenolpyruvate carboxylase. Our results show that complete expression of the glyoxylate shunt results in higher extracellular succinate accumulation compared to the wild type control strain after incubation of cells in darkness and anoxic darkness in the presence of nitrate. Addition of the inhibitor 2-thenoyltrifluoroacetone increased succinate titers in all the conditions tested when nitrate was available. Addition of acetate in the presence of the inhibitor further increased the succinate accumulation, resulting in high levels when phosphoenolpyruvate carboxylase was overexpressed, compared to control strain. However, the highest succinate titer was obtained after dark incubation of an engineered strain with a partial glyoxylate shunt overexpressing isocitrate lyase in addition to phosphoenolpyruvate carboxylase, with only 2-thenoyltrifluoroacetone supplementation to the medium.

**Conclusions:**

Heterologous expression of the glyoxylate shunt with its central link to the tricarboxylic acid cycle (TCA) for acetate assimilation provides insight on the coordination of the carbon metabolism in the cell. Phosphoenolpyruvate carboxylase plays an important role in directing carbon flux towards the TCA cycle.

## Background

Cyanobacteria are the oldest photosynthetic organisms on Earth and were responsible for raising O_2_ levels when the atmosphere was anoxic [1–3]. Today, cyanobacteria are receiving increased attention as photosynthetic microbial cell factories [4–6]. They are relatively easy to grow and produce valuable native substances such as fatty acids and terpenoids, among others [7, 8]. In addition, they can be genetically engineered to produce non-native molecules of human interest such as ethanol, butanol, mannitol, isobutyraldehyde, isobutanol, isoprene, ethylene [9–15].

Succinate is an important industrial chemical as a precursor of 1,4-butanediol, adipic acid and other four-carbon chemicals. It can also be used for the production of pharmaceuticals, antibiotics, and amino acids [16, 17]. Succinate is a key metabolite in the tricarboxylic acid cycle (TCA cycle) and it is produced naturally by many microorganisms through fermentation [18–22]. Cyanobacteria are able to secrete succinate, and cyanobacteria genetically engineered for succinate production have received attention in the last years [23–25].

**Fig. 1:**
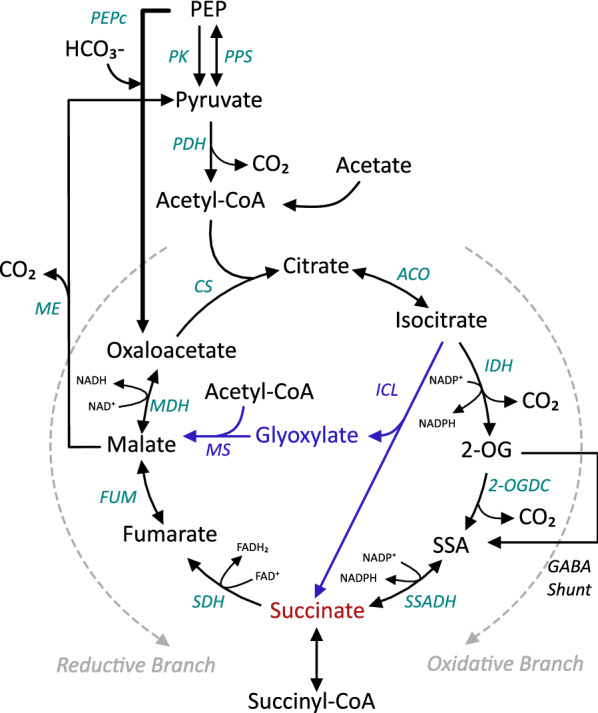
Carbon metabolism involved in the TCA cycle of *Synechocystis* PCC 6803. Glyoxylate shunt introduced in the engineered *Synechocystis* cells indicated in blue color. Abbreviations: 2-OG, 2-oxoglutarate; 2-OGDC, 2-oxoglutarate decarboxylase; ACO, aconitase; CS, citrate synthase; FUM, fumarase; GABA, γ-aminobutyrate; ICL, isocitrate lyase; IDH, isocitrate dehydrogenase; MDH, malate dehydrogenase; ME, malic enzyme; MS, malate synthase; PDH, pyruvate dehydrogenase complex; PEP, phosphoenolpyruvate; PEPc, phosphoenolpyruvate carboxylase; PK, pyruvate kinase; PPS, phosphoenolpyruvate synthase; SDH, succinate dehydrogenase; SSA, succinic semialdehyde; SSADH, succinic semialdehyde dehydrogenase

The TCA cycle is central in cyanobacterial metabolism. During light conditions, when photosynthesis is active and the cells produce biomass, the cycle plays an important role by providing carbon skeletons for the nitrogen metabolism via 2-oxoglutarate (2-OG) and synthesis of glutamate family amino acids, and aspartate amino acids synthesis via oxaloacetate (OAA). In this case, both the oxidative and reductive directions of the TCA cycle are active (Fig. 1) [26, 27]. In darkness and in the presence of oxygen, succinate dehydrogenase (SDH) plays an important role by providing electrons to the respiratory electron chain and therefore the oxidative branch of the TCA cycle is the most active. In contrast, when the cells are fermenting they need to remove excess electrons and therefore succinate is secreted (together with other compounds such as lactate and acetate) [28].

Many bacteria and some cyanobacteria possess the glyoxylate shunt [29–32]. The glyoxylate shunt operates in two steps (Fig. 1). The first enzyme of the shunt, isocitrate lyase (ICL), cleaves isocitrate to succinate and glyoxylate. The latter is condensed with acetyl-CoA by a second enzyme, malate synthase (MS), to form malate, which in turn can be oxidized to OAA and converted into PEP via gluconeogenesis. The net reaction is the formation of one four-carbon dicarboxylic acid from two molecules of acetyl-CoA, while bypassing two carbon dioxide releasing decarboxylation steps of TCA cycle (Fig. 1). The glyoxylate pathway is essential for growth on substrates such as acetate, fatty acids and other carbon sources that degrade exclusively to acetyl moieties. In this case, the bypass conserves some of the carbon to generate glucose (and hence, biomass production) through gluconeogenesis. However, in *Synechocystis*, the first enzyme in the gluconeogenetic pathway, PEP carboxykinase (PEPCK), that converts OAA into PEP is absent [33].

The role of glyoxylate shunt that was discovered in some cyanobacteria remains unclear, since cyanobacteria are ubiquitous photoautotrophs with CO_2_ being the main carbon source. It is suggested, that some of the nitrogen fixing cyanobacteria, which naturally possess a glyoxylate shunt belong to a special ecological niche where acetate is as least temporarily present and supports assimilation of nitrogen or organic compounds during night phase [33].

An essential enzyme for cyanobacteria that is involved in the TCA cycle is phosphoenolpyruvate carboxylase (PEPc) [34]. PEPc catalyzes the irreversible reaction of PEP and bicarbonate to produce OAA and inorganic phosphorous. The anaplerotic reaction of PEPc is a main route to replenish OAA in the TCA cycle, since OAA is constantly depleted for the synthesis of aminoacids [35]. The overexpression of this enzyme has been shown to lead to an increased growth at low light, and higher photosynthetic activity in standard conditions as well as when environmental stresses are present in cyanobacteria [36, 37].

Several studies have been published where cyanobacteria were engineered in order to increase the succinate production [15, 23–25, 27, 28, 38–41]. In the cyanobacterium *Synechococcus elongatus* PCC 7942, the heterologous overexpression of citrate synthase (CS), 2-oxoglutarate decarboxylase, succinate semialdehyde dehydrogenase and PEPc increased succinate production when cultivated in light [23]. In another study, the authors introduced a CRISPRi based knock down of the glycogen synthesis gene while overexpressing CS and PEPc [39] resulting in higher succinate secretion when the cells were cultivated in light and nitrogen starvation. Moreover, deletion of SDH in *Synechocystis* boosted succinate yields [25, 41].

The aim of this study was to investigate the effect on extracellular succinate accumulation of introducing ICL alone or in an operon with MS, both from *Escherichia coli* BL21, into an engineered strain of *Synechocystis* containing two additional copies of the native phosphoenolpyruvate carboxylase [37]. The action of ICL may direct more carbon towards the formation of succinate, thus possibly increasing succinate accumulation in the media, whereas MS contributes to closing the glyoxylate bypass. Extracellular succinate accumulation was investigated and compared after incubation of different engineered strains of *Synechocystis* under a range of different environmental conditions.

## Results

### Engineered strains

In previous work, an engineered strain of *Synechocystis* that overexpresses PEPc was obtained [37]. This strain, called WT + 2xPEPc, was used as a background strain for integration of the glyoxylate shunt in *Synechocystis*, along with the control strain WT + Km^r^, which contains only an antibiotic resistance cassette [37]. Two enzymes from *E. coli*, ICL and MS, encoded by *aceAB*, were selected for expression in *Synechocystis* in order to construct a glyoxylate shunt in this organism. *aceA* alone as well as *aceAB* as an operon were integrated in the *Synechocystis* genome and expressed from the Ni^2+^-inducible promoter P*nrsB* [42]. We also constructed two control strains, WT_C and 2P_C, where only a resistance cassette was integrated instead of the *aceAB* expression construct (Table 1).

**Table 1:** Engineered strains of *Synechocystis* PCC 6803 used/created in this study

Engineered strain	Genotype	Reference
WT + Km^r^	*Synechocystis* 6803 Δ*psbA2*::*Km*^*r*^	Durall et al. [37]
WT + 2xPEPc	*Synechocystis* 6803 Δ*psbA2*::*pepC-Km*^*r*^* pepC*-*pepC*	Durall et al. [37]
WT_C	*Synechocystis* 6803 Δ*psbA2*::*Km*^*r*^* slr1068*::*Cm*^*r*^	This study
2P_C	*Synechocystis* 6803 Δ*psbA2*::*pepC-Km*^*r*^* pepC*-*pepC slr1068*::*Cm*^*r*^	This study
2P_I	*Synechocystis* 6803 Δ*psbA2*::*pepC-Km*^*r*^* pepC*-*pepC slr1068*:: P*nrsB-aceA*_*Ec*_-*Cm*^*r*^	This study
2P_IM	*Synechocystis* 6803 Δ*psbA2*::*pepC-Km*^*r*^* pepC*-*pepC slr1068*:: P*nrsB-aceAB*_*Ec*_-*Cm*^*r*^	This study

When the control strain WT + Km^r^ was transformed to express the complete glyoxylate shunt, encoded by *aceAB*, no colonies could be obtained. However, it was possible to transform the control strain to express only ICL, although this strain was not further evaluated in this study. The strain WT + 2xPEPc could be transformed with the construct carrying *aceAB* plasmid to form the strain 2P_IM, and with the contruct for ICL alone, creating the 2P_I strain. The engineered strains were subsequently used to investigate accumulation of succinate in the media under different conditions. For an overview of strains used in this study, see Table 1.

### PEPc, ICL, and MS protein levels

**Fig. 2:**
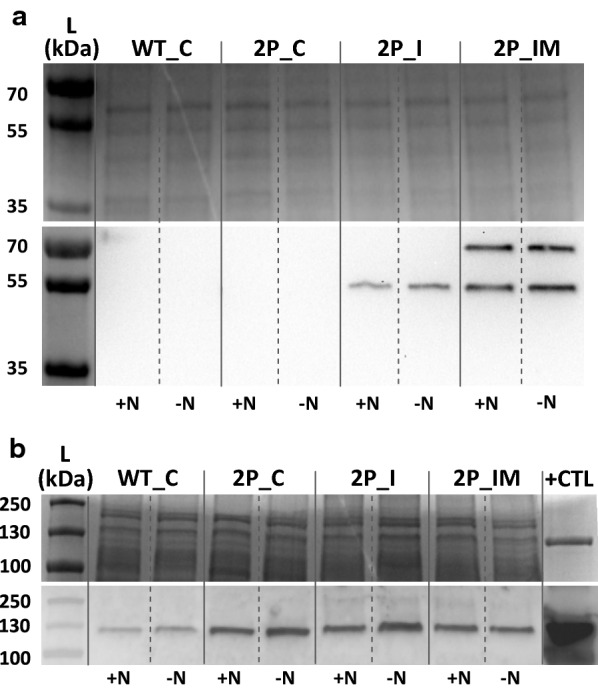
SDS-PAGE and Western immunoblot on extracts of the *Synechocystis* strains created in this study. SDS-PAGE/Western immunoblot analysed for the presence of ICL and MS (**a**); analysed for the presence of PEPc (**b**). **a** Upper panel shows SDS-PAGE loaded with 5 µg of protein crude extract from the *Synechocystis* engineered strains created in this study (Table 1). Lower panel shows Western immunoblot using anti-Strep antibody for the *Synechocystis* engineered strains (Table1). Bands correspond to MS (approximate molecular weight 60 kDa) and to ICL (approximate molecular weight 47 kDa). Samples are from cells incubated under darkness. **b** Upper panel shows SDS-PAGE loaded with 31 µg of protein crude extract from *Synechocystis* strains created in this study (Table 1). Lower panel displays Western immunoblot using anti-PEPc for the *Synechocystis* engineered strains. The approximate molecular weight of PEPc is 110 kDa. Control (CTL) corresponds to purified PEPc from the cyanobacterium *Synechocystis* PCC 6803. Samples are from cells incubated under anoxic darkness

The presence of the protein ICL was successfully detected by Western immunoblots in the engineered strains overexpressing PEPc and the partial or complete glyoxylate shunt (2P_I and 2P_IM), while the presence of MS was only detected in the strain 2P_IM (Fig. 2a). The control strain without overexpressed PEPc (WT_C) showed lower PEPc protein content compared to the engineered strains overexpressing PEPc (2P_C, 2P_I and 2P_IM) (Fig. 2b).

### Succinate accumulation from cells incubated in light

**Fig. 3:**
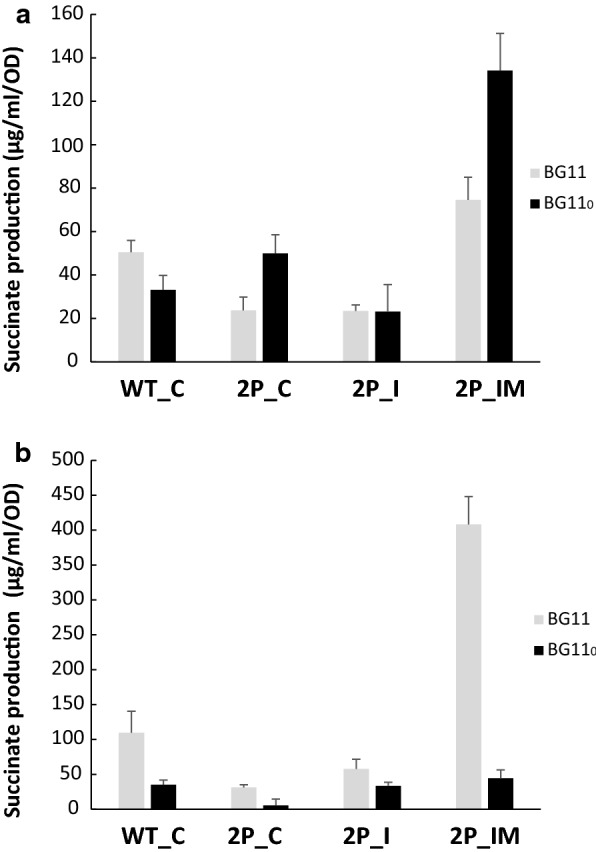
Succinate titers in the growth medium of *Synechocystis* strains created in this study in light and darkness. Cells were grown in pre-cultures, then concentrated and incubated for four days in 20 µE m^−2^ s^−1^ light (**a**) or in darkness (**b**), before determination of succinate titers. BG11 and BG11_0_ corresponds to media with nitrate or without any nitrogen source, respectively. 5 µM of NiCl_2_ was added for induction of gene expression. Error bars represent the mean ± SD

**Fig. 4:**
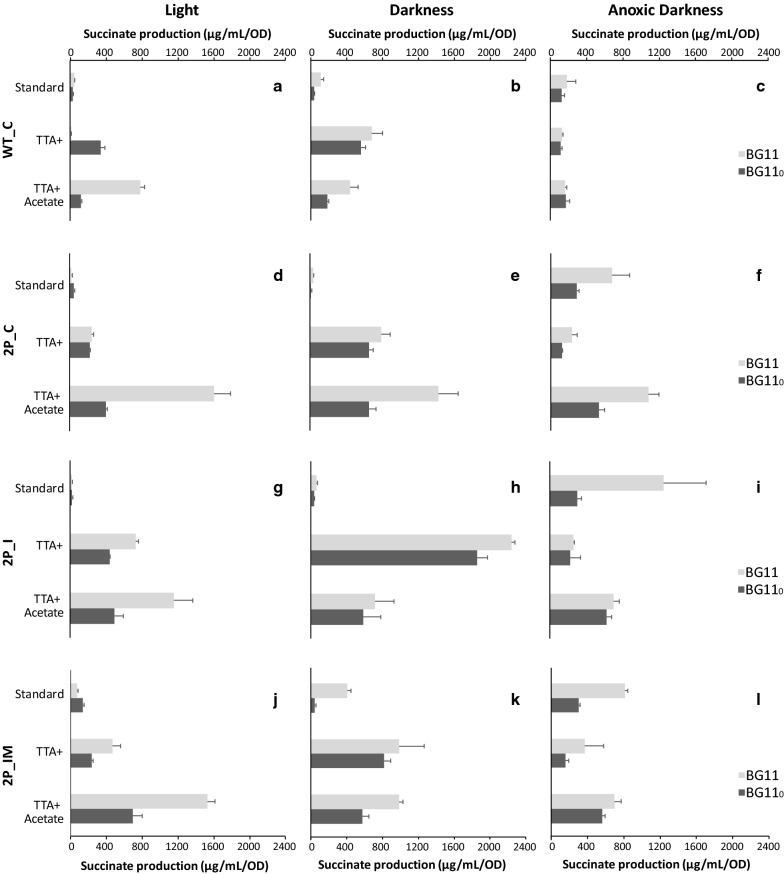
Succinate titers in the growth medium of *Synechocystis* strains created in this study under all tested conditions. BG11 corresponds to media with nitrate, BG11_0_ corresponds to media without any nitrogen source. Standard corresponds to BG11 or BG11_0_ and 5 µM of NiCl_2_ incubated for 4 days. + TTA corresponds to BG11 or BG11_0_, 5 µM of NiCl_2_ and 1 mM of 2-thenoyltrifluoroacetone (TTA) incubated for 4 days. TTA + Acetate corresponds to BG11 or BG11_0_, 5 µM of NiCl_2_, 1 mM of TTA, 50 mM of Tris pH 7.5 and 0.2% acetic acid glacial incubated for 4 days. Error bars represent the mean ± SD

After cultivation in bubbling flasks under light until the OD_750_ reached 1, the cells were concentrated and resuspended into BG11 or BG11_0_ in 6-well plates with required antibiotics and addition of NiCl_2_ for induction of expression of the genes encoding the glyoxylate shunt. The plates were incubated with constant shaking at 30 °C. Succinic acid excreted into the medium was analysed by HPLC–ESI–MS. After 4 days of incubation, succinate was detected in growth media of all engineered strains regardless of the presence or absence of nitrate (Figs. 3a and 4a, d, g, j). The quantification of succinate and the statistical analysis are summarized in Additional files 1, 2, 3, 4, and 5, respectively (see Additional files 1, 2, 3, 4, and 5).

Under photosynthetic, nitrogen replete conditions, the engineered strains overexpressing PEPc alone or in combination with expression of the partial glyoxylate shunt (2P_C and 2P_I) showed two fold lower succinate accumulation compared to the WT_C strain (Figs. 3a and 4a, d, g), whereas the accumulation of succinate by the engineered strain overexpressing PEPc with a complete glyoxylate shunt (2P_IM) was similar to the control strain WT_C (Figs. 3a and 4a, j).

When the cells were grown under nitrogen starvation condition in light, higher succinate accumulation was observed only for strains 2P_C and 2P_IM, with one and a half and four times higher levels compared to control strain WT_C, respectively. In addition, no bleaching was observed after the incubation in nitrogen deplete media, for any of the strains in all tested conditions.

### Succinate accumulation from cells incubated in darkness

Cultures of *Synechocystis* were treated in the same way as during the light experiments with the difference that immediately after addition of Ni^2+^ inducer, the 6-well plates were covered with aluminium foil. Levels of succinate in the media after 4 days of incubation in darkness were increased compared to the light condition, in the presence of nitrate (Figs. 3b and 4a, b, d, e, g, h, j, k). In contrast, for all strains the absence of combined nitrogen in the media resulted in similar or lower succinate levels than in light. The strain 2P_IM (Figs. 3b, 4k) accumulated 10 times more succinate in nitrogen replete media than after incubation in the media without any nitrogen source during the same experimental condition.

Comparing between strains, the engineered strains 2P_C and 2P_I showed similar succinate titers, lower than the control strain, when the cells were incubated in the dark with nitrate (Figs. 3b and 4b, e, h). However, 2P_IM showed almost four times higher succinate titers compared to WT_C (Figs. 3b and 4h, k). When the cells were cultivated under nitrogen starvation conditions, similar levels of accumulated succinate were observed among the strains WT_C, 2P_C, 2P_I and 2P_IM (Figs. 3b and 4b, e, h, k).

### Succinate accumulation from cells incubated in dark anoxic conditions

For the anoxic darkness experiments, cells were incubated in anaerobic conditions in sealed vials in the dark. All engineered strains showed higher succinate titers after 4 days of incubation under these conditions, compared to light and darkness (Fig. 4c, f, i, l). In the control strain WT_C under anoxic darkness, the levels of succinate were similar when the cells were incubated in the presence or absence of nitrate (Fig. 4c). For the strains 2P_C, 2P_I and 2P_IM, less succinate was accumulated when the cells were incubated in nitrogen starvation compared to nitrogen replete conditions (Fig. 4f, i, l).

In the presence of nitrate, 2P_C, the engineered strain overexpressing PEPc alone, showed three times higher succinate titer compared to the control strain WT_C (Fig. 4c, f). The strain with the complete glyoxylate shunt and PEPc overexpression, 2P_IM, accumulated four times more succinate in the media compared to the WT_C (Fig. 4c, l). The 2P_I strain, with only half of the glyoxylate shunt, showed the highest succinate titer (5.5 times higher than WT_C) in anoxic darkness and presence of nitrate (Fig. 4i).

### Succinate accumulation in the presence of TTA

In order to test if inhibition of succinate dehydrogenase (SDH) would increase succinate titers, the inhibitor 2-thenoyltrifluoroacetone (TTA) was added to the media after concentrating the cells. Increased succinate accumulation in the medium was observed (compared to no addition of TTA) in all the conditions for all the strains used in this study (Fig. 4), with the exception of anoxic darkness, and for the WT_C and 2P_IM strains during light and dark incubations, respectively. The control strain WT_C showed approximately three times lower succinate levels in the media with addition of TTA in comparison to standard nitrogen replete conditions in light (Fig. 4a).

In light and in presence of nitrate, 16 times more succinate accumulated comparing 2P_C to the control strain WT_C (Fig. 4a, d). In 2P_I and 2P_IM, succinate titers increased approximately 48 and 30 times, respectively compared to WT_C (Fig. 4d, j). No significant differences in succinate accumulation were observed between the strains compared to WT_C when strains were incubated in light with nitrogen starvation and addition of TTA (Fig. 4a, d, g, j).

In darkness, TTA treatment led to three times higher succinate accumulation in the media in 2P_I compared to in WT_C (Fig. 4b, h) regardless of the presence or not of nitrate. In nitrogen replete media this strain reached the maximal detected concentration of all the tested strains and conditions. For all strains, nitrogen starvation conditions with addition of TTA showed slightly lower levels of succinate than in nitrogen replete media with TTA (Fig. 4b, e, h, k).

In anoxic darkness with TTA, and in both presence and absence of nitrate, the control strain WT_C showed similar levels of succinate compared to when SDH was not inhibited (Fig. 4c). Interestingly, for all strains except the control, inhibition of SDH led to lower succinate levels than in standard condition with nitrogen available.

Double amount of succinate was detected for the engineered strain 2P_I compared to WT_C (Fig. 4c, i) when the cells were grown in anoxic darkness with nitrogen in the media and TTA treatment. Conversely, in nitrogen deplete conditions no differences in succinate titers were found among strains.

### Succinate accumulation in the presence of TTA and acetate

In order to test if higher succinate titer could be achieved when acetate is present, the cells after concentration were incubated with 1 mM of TTA and 0.2% of acetate. In the light, this treatment in combination with growth in nitrogen replete medium resulted in higher extracellular succinate accumulation for all strains except 2P_I (Fig. 4a, d, j).

Comparing between the strains in light and with nitrate, 2P_C and 2P_IM showed increased succinate titers, with almost one and a half and two times more, respectively, compared to WT_C (Fig. 4a, d, j). When nitrogen was not available, the strains overexpressing PEPc, 2P_C, 2P_I and 2P_IM, showed increased succinate levels in the medium compared to the control strain by three, four and five times, respectively (Fig. 4a, d, g, j).

When cells were treated with TTA and acetate in the dark and in the presence of nitrogen, 2P_IM showed more than two times higher succinate titer compared to the control strain WT_C, while 2P_C showed three times more. In darkness and nitrogen starvation, the same strains, 2P_C and 2P_IM, showed three times increase in detected extracellular succinate in comparison to control strain (Fig. 4b, e, k). Notably, for strain 2P_I no increase in succinate level was observed after the treatment with TTA and acetate in dark both with and without nitrate compared to WT_C, unlike what was observed with TTA but without addition of acetate.

In anoxic darkness with nitrogen availability upon combined treatment with TTA and acetate, the strains with partial or complete glyoxylate shunt, 2P_I and 2P_IM, increased succinate titers more than four times compared to WT_C, while for the strain 2P_C that only overexpressed PEPc six times more succinate compared to WT_C was detected in the media. In anoxic darkness and nitrogen starvation, no significant changes in succinate accumulation in the media were observed between strains compared to control strain WT_C (Fig. 4c, f, i, l).

### Cell growth under different conditions

**Fig. 5:**
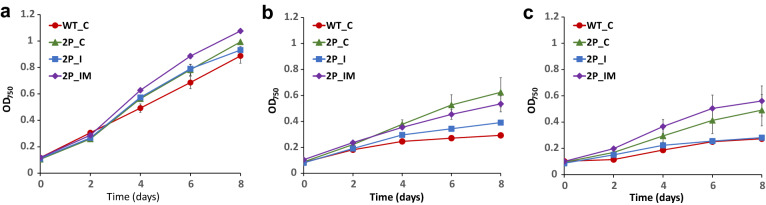
Growth of the *Synechocystis* strains under different conditions. **a** Growth under light. The cells were cultivated under 20 μmol photons m^−2^ s^−1^ for 8 days and induced with 2.5 µM of NiCl_2_ every second day. **b** Growth under light with acetate. The cells were cultivated as for (A), with addition of 0.2% acetate every second day. **c** Growth in darkness with acetate. The cells were cultivated in the dark with addition of 2.5 µM of NiCl_2_ and 0.2% acetate every second day during 8 days. Error bars represent the mean ± SE

The engineered strains overexpressing PEPc (2P_C, 2P_I and 2P_IM) showed higher growth rates compared to the control strain (WT_C) under standard light conditions (p = 0.04) (Fig. 5). However, no significant differences were observed in the growth rate among all the engineered strains overexpressing PEPc (2P_C, 2P_I and 2P_IM) (Fig. 5a). No significant differences were observed in growth when the strains were grown in the presence of 50 mM Tris, pH 7.5 compared to the absence of buffer (data not shown).

When cultivated in the light with acetate, the engineered strain expressing the partial glyoxylate shunt and overexpressing PEPc (2P_I) did not show significant differences in growth rate compared to the control strain (WT_C) (Fig. 5b). Interestingly, 2P_C and 2P_IM both grew slightly better than the control WT_C and 2P_I (Fig. 5b).

In darkness with addition of acetate, the growth rates of the engineered strains overexpressing PEPc alone (2P_C) or expressing the complete glyoxylate shunt (2P_IM) were higher than that of the control strain (WT_C) (p = 0.04 and p = 0.003, respectively) (Fig. 5c). However, no significant differences were detected between the 2P_IM and 2P_C. As observed in cells grown in light with acetate, 2P_I did not show significant differences in growth rate compared to the control strain (WT_C) (Fig. 5c).

### Discussion

In cyanobacterial cells, the TCA cycle reactions provide reducing power and essential precursor metabolites. In this study, we aimed to introduce a glyoxylate shunt in the TCA cycle in a strain of the cyanobacterium *Synechocystis* PCC 6803 already engineered to overexpress PEPc, and investigate whether this would have an effect on excretion of succinate from the cells under different conditions.

We found that expression of the glyoxylate shunt in *Synechocystis* seems to have strong impact on the cell metabolism, since it was not possible to obtain wild type *Synechocystis* expressing both ICL and MS genes, but only the partial shunt expressing ICL. It is known that the P*nrsB* promoter has a basal level of expression in BG11 medium, without induction with extra Ni^2+^ [42], and likely the minimum expression of enzymes affects the viability of the cells.

A competition by ICL with isocitrate dehydrogenase (IDH) for substrate may have negative effect on the cells, however, the affinity of purified ICL from *E. coli* for substrate is higher than that determined for from *Synechocystis* IDH [43, 44]. Therefore, flux through IDH would be favoured, especially at low carbon flux in the TCA cycle, as in WT *Synechocystis*. Heterologous expression of MS might create a lethal phenotype due to consumption of one of its substrates, and malate synthase from *E. coli* has quite high affinity for both substrates [45]. However, acetyl-CoA that is consumed by heterologous MS is recirculated via the malic enzyme into pyruvate [46]. The second substrate for MS, glyoxylate, is normally produced via oxidation of 2-phosphoglycolate (2PG), a product of photorespiration [47, 48], and a detrimental effect of expression of MS on the cells may be due to either interfering with the use of glyoxylate for generation of glycine and serine, or have to do with the role of 2PG being a reporter molecule in signalling mechanism of carbon/nitrogen ratio in *Synechocystis* [49]. Either way, overexpression of PEPc relieved the effect on cell metabolism, possibly because elevation of OAA levels available for the TCA cycle allows the partitioning of isocitrate towards glyoxylate and malate, and thus even when MS is expressed the glyoxylate pool is not drained.

In case of partial glyoxylate shunt expression, strain 2P_I, it is plausible that glyoxylate, co-synthesised with succinate, would be incorporated into glycine aminoacid pool. The flux through other photorespiratory routes are suggested to be minimal in *Synechocystis* [50, 51].

In light and BG11 media, however, overexpression of PEPc and the introduction of genes for the glyoxylate shunt did not significantly enhance succinate accumulation in the media. After addition of the SDH inhibitor TTA, levels of succinate in the media increased in all engineered strains compared to WT_C. It has been shown that SDH is responsible for the main donation of electrons to the respiratory electron transport chain [52], and accumulation of succinate after SDH inhibition provides evidence of the amount of carbon flow into the cycle. In a previous study, deletion of SDH led to significant succinate accumulation in the media during the stationary growth phase [41]. However, in another study, during mid-exponential growth phase, an SDH deletion mutant accumulated less succinate than the wild type [53].

In our study, the higher succinate levels detected after TTA addition in all engineered strains compared to the WT_C control may be due to higher carbon flow into the TCA cycle with the extra copies of PEPc, providing extra OAA to the cycle. A lower concentration of PEP due to the action of PEPc may also relieve inhibition of citrate synthase (CS) by PEP, which has been shown to be a feature of *Synechocystis* CS [54]. This would result in higher CS activity at the same time as PEPc provides more OAA substrate, thus further acting to increase TCA cycle activity. Interestingly, partial and complete glyoxylate shunt strains accumulated significantly higher levels of succinate compared to the 2P_C strain, also possibly due to directly increased carbon flow towards succinate production.

It is known that depletion of nitrogen in the growth media causes a cascade of changes in the cell metabolism, promoting the synthesis of glycogen and finally entering a dormant state. Many studies assume that the nitrogen status of the cell is based on the sensing of the reporter molecule 2-oxoglutarate, an intermediate of the TCA cycle [49]. Contrary to a previous report for *Synechococcus elongatus* PCC 7942 [39], succinate accumulation in *Synechocystis* cultures did not increase during stationary phase in nitrogen-free media in any tested condition compared to nitrogen replete media, except for the WT_C strain with addition of TTA. This is in agreement with a previous study [55], where during 7 days prolonged nitrogen starvation of mid-exponential grown *Synechocystis* cells, levels of succinate remained almost unchanged.

Interestingly, while comparing the engineered strains to WT_C under nitrogen limitation in light and presence of TTA, all strains including WT_C showed a similar level of succinate production, indicating that carbon flux through the TCA cycle was not altered by PEPc overexpression with or without the glyoxylate shunt compared to the control strain. As suggested by Woude et al. [56], during nitrogen deprivation carbon flow towards pyruvate is restricted and redirected to glycogen synthesis. This consequently leads to less PEP available for the PEPc reaction, and the flux to OAA via PEPc, which boosts succinate production, is thus limited by substrate availability. Blocking the glycogen synthesis has been shown to result in an increased succinate production during nitrogen starvation [39].

Succinate accumulation in the media was further increased in engineered strains compared to WT_C after the addition of acetate and TTA to the growth media with and without nitrogen. The growth of the engineered strains was affected by the presence of acetate, since the cultures in the light without acetate addition, reached higher OD. Similarly to the observations made in *Synechococcus* PCC 7002 with *aceBA* genes from *Chlorogloeopsis fritschii* PCC 9212 [31], which has a native glyoxylate shunt, we did not observe increased growth of the engineered strain with the complete glyoxylate shunt (2P_IM) in darkness and with acetate as an additional carbon source. Potentially it might be due to absence of PEPCK in both *Synechocystis* and *Synechococcus* PCC 7002.

By the addition of acetate to growth media, the acetyl-CoA pool is expected to increase and provide an additional carbon source for biomass building blocks [57]. Increased intracellular acetate may limit pyruvate decarboxylation reaction by reducing the CoA pool for the formation of acetyl-CoA [58], which may lead to increased phosphoenolpyruvate availability for the PEPc reaction. This would explain why succinate titers were higher among the engineered strains 2P_C and 2P_IM compared to the wild type control strain WT_C after addition of acetate and TTA in light. However, under the same conditions no difference was observed in succinate production between the PEPc overexpressing strain 2P_C and the partial or complete glyoxylate shunt strains 2P_I or 2P_IM. Together with the data on growth on acetate in low light, these results indicate that the expression of the glyoxylate shunt in addition to the overexpression of PEPc in *Synechocystis* did not improve the assimilation of acetate. The anaplerotic reaction of PEPc seems to play a main role in directing carbon flux into the TCA cycle. Presumably, uptake of acetate is restricted by its transport inside the cell, as indicated [57], and overexpression of an acetate transporter may lead to better acetate incorporation.

Upon shifting the environment into darkness, the survival of the cells depends on stored carbon compounds, since there is no photosynthetic supply of acetyl-CoA. Here, blocking of SDH, regardless of the presence of nitrate, led to much higher succinate production than in light, showing that the TCA cycle is active in the oxidative direction in order to produce succinate, later used by SDH to donate electrons to the respiratory electron transport chain to drive respiration.

In darkness, overexpression of PEPc did not result in an increased succinate accumulation, probably due to lower levels of PEP [38, 59] and HCO_3_^−^ [60]. However, in combination with expression of the complete glyoxylate shunt, in strain 2P_IM in comparison to WT_C, more succinate accumulation was observed, probably due to the operation of glyoxylate shunt, which leads to succinate being formed in excess of what is needed by SDH under dark conditions.

In our study, heterologous overexpression of only ICL, without MS, in *Synechocystis* may create a shortcut towards succinate, which would work as a carbon sink. ICL consumes some of the isocitrate pool by competing with IDH, whereas there is still a pull of downstream metabolites which need to be replenished to their normal levels (mainly 2-oxoglutarate, as it is constantly removed for nitrogen assimilation). Subsequently, this may result in an upstream upregulation to fulfil the increased demand of citrate and other precursors, leading in the end to the double amount of succinate observed in strain 2P_I compared to strains 2P_C or 2P_IM, after addition of TTA. In other words, in order to withstand a depletion of downstream metabolites by cause of heterologous ICL and survive, cells need to synthesise more of the upstream metabolites, which in the end leads to increased succinate formation. Effectively, this creates a link between growth of the cells and the generation of the desired product succinate.

In case of strain 2P_IM with a complete glyoxylate shunt this sink effect on carbon flux disappears, possibly due to a restored balanced carbon partitioning in the TCA cycle. In this scenario, for each isocitrate molecule used by heterologous expressed ICL, malate is synthesized by MS from glyoxylate in addition to the succinate formed by ICL. Thus, an equimolar concentration of malate replenishes the pool of precursors for TCA cycle operation, and the demand of carbon precursors for citrate and isocitrate synthesis may remain as in WT or 2P_C. Probably, the reason why this sink effect on carbon flux is observed in darkness is that in the dark, the cyclic action of the TCA cycle is more important [52]. Addition of acetate is expected to increase the pool of acetyl-CoA, and can provide a high enough abundance of metabolites and therefore this possible “sink effect” and the subsequent upregulation for the precursors is not created in 2P_I strain when grown with acetate addition.

It has been previously shown that overexpression of PEPc led to an increased succinate production in dark anoxic conditions in the presence of nitrate in *Synechocystis* [27]. In dark anoxic conditions, it has been shown that the reductive branch of the TCA cycle is the most active [27]. However, some carbon flux is present towards isocitrate of the oxidative branch, since the PEPc overexpressing strains engineered to express the complete glyoxylate shunt (2P_IM) showed higher succinate production compared to the WT_C when nitrate was available.

Interestingly, after the addition of TTA to the media, less succinate was accumulated during anoxic darkness than in standard condition, probably due to inhibition of succinate formation and suggesting that succinate originated from fumarate. This data is in agreement with the results of metabolite turnover analysis [27], which showed that 2-oxoglutarate had the lowest ^13^C fraction among the metabolites of the TCA cycle, indicating that succinate is forming mostly from fumarate during dark anoxic conditions. Furthermore, up to date is it rather unclear if the action of SDH complex can be reversible, meaning if it can also perform reduction of fumarate. Early investigation on the role of SDH in the respiratory electron transport chain in *Synechocystis* suggested no fumarate reductase activity [52, 53]. It can also be noted that available studies, including recent ones [41], were performed by the deletion of open reading frames of SDH subunits. Explicit in vitro studies of SDH were not performed. Our data, together with metabolite turnover flux analysis [27], supports succinate formation from fumarate in dark anoxic conditions. A possible alternative candidate for the fumarate reduction in *Synechocystis* might be L-aspartate oxidase (EC:1.4.3.16; sll0631) encoded by the putative *nadB* gene. In *E. coli* this enzyme (Uniprot entry P10902) can oxidase aspartate by using oxygen or fumarate as electron acceptors forming iminosuccinate, peroxide or succinate subsequently. A study on crystal structures of L-aspartate oxidases indicates a high similarity to succinate dehydrogenases/fumarate reductases [61], which may result in a similar inhibitory effect of TTA as for the SDH. In addition, the possibility of the existence of another route to synthesize succinate by the action of L-aspartate oxidase in cyanobacteria was noted in [62]. Their quadruple knock out mutant of *Synechococcus* PCC 7002 which lacks all enzymes known to form succinate, namely SDH, succinyl-CoA synthase (SucCD), succinic semialdehyde dehydrogenase (SSADH) and 2-oxoglutarate decarboxylase (2-OGDC), still accumulated similar levels of succinate as the wild type strain.

## Conclusions

In this study, we aimed to produce succinate by expressing a glyoxylate shunt that is missing in *Synechocystis*. Firstly, we were not able to introduce genes for the glyoxylate shunt from *E. coli* in the WT + Km^r^, but only when the strain with overexpressed PEPc was used as a background strain. The extracellular succinate accumulation of control strain 2P_C was similar to that of the glyoxylate shunt strain 2P_IM in light and standard media, indicating that while the anaplerotic reaction of PEPc plays an important role in directing carbon flux towards the TCA cycle, heterologous expression of the glyoxylate shunt genes did not further increase succinate titer in the medium under these conditions. The partial introduction of the glyoxylate shunt resulted in the highest succinate titer achieved in this study, during darkness and addition of TTA, but had no improvement of growth compared to the wild type control strain. In future experiments, it would be interesting to further engineer 2P_I by knocking out or downregulating SDH, mimicking the effect of TTA addition. The resulting strain would be interesting to cultivate in diurnal cycles, since in our experiments, the corresponding cells showed increased succinate accumulation in both light and dark conditions. The findings reported above indicate the importance of understanding the regulation of carbon metabolism, particularly the TCA cycle and glycogen turnover, for successful engineering of phototrophic cyanobacteria for succinate production.

## Material and methods

### Bacterial strains and growth conditions

*Escherichia coli* strain DH5α was used for subcloning according to the standard procedures. *E. coli* cells were grown in LB medium, with addition of antibiotics as appropriate Kanamycin (Km) (25 µg mL^−1^) or Chloramphenicol (Cm) (20 µg mL^−1^). *Synechocystis* WT + Km^r^ and WT + 2xPEPc cells were cultivated in BG11 medium [1] and Km (25 µg mL^−1^).

For the growth experiments, the engineered strains were grown in light (20 µE m^−2^ s^−1^) with BG11 containing Km (25 µg ml^−1^), Cm (20 µg ml^−1^) and 2.5 µM of NiCl_2_ or in light and darkness with BG11 containing 50 mM Tris pH 7.5, Km (25 µg ml^−1^), Cm (20 µg ml^−1^), 0.2% of glacial acetate and 2.5 µM of NiCl_2_. Every second day the OD_750_ was measured using the Varian 50 Bio UV–Visible Spectrophotometer and NiCl_2_ (final concentration 2.5 µM) was added. When the media contained acetate, it was added (final concentration of 0.2%) every second day. At least two experiments were done independently with three biological replicates each. The growth rate was then calculated by plotting the log of the OD_750_ values versus time (8 days). The slopes obtained were used to perform the statistical analysis, Student's two-tailed *t*-test, p < 0.05.

### Construction of plasmids

Genes encoding ICL (encoded by *aceA*, accession number P0A9G6) and MS (encoded by *aceB*, accession number P08997) were cloned using *Escherichia coli* BL21 genomic DNA as a template. The *aceA* was amplified with oligos oligoF-AXS and oligoR-ABP and inserted with N-terminus strep-tag into pEERM3 + , a derivative of the vector pEERM3 [63] under P*nrsB* promoter using *XbaI*-site directly after RBS* [64] and *PstI*-site further downstream creating pEERM3-aceA. *aceB* was amplified using oligos oligoF-BBRS and oligoR-BP and inserted into pEERM3-aceA downstream of *aceA* with synthetic RBS* and N-terminus strep-tag using *BglII* and *PstI* sites to create pEERM3-aceAB. Plasmids were sequenced to confirm correct insertion. The sequence of the primers and plasmids created are summarized in Table 2.

**Table 2:** Plasmids and oligonucleotides used/created in this study

Primer/ Plasmid	Sequence/Construct
oligoF-AXS	TATGTCTAGAATGTGGAGTCATCCTCAGTTCGAGAAGGGTAGCGGAAGTAAAACCCGTACACAACAAAT
oligoR-ABP	TGAACTGCAGTAACTTAGATCTTTAGAACTGCGATTCTTCGG
oligoF-BBRS	CTTAAGATCTTAGTGGAGGTAAGCTTATGTGGAGTCATCCTCAGTTCGAGAAGGGTAGCGGAAGTACTGAACAGGCAACAACAACC
oligoR-BP	TGAACTGCAGTTACGCTAACAGGCGGTAGC
pEERM3 +	Plasmid (empty backbone) for integration into neutral site of *Synechocystis* 6803, based on the pJ344 vector backbone, carrying chloramphenicol resistance cassette, P*nrsB* promoter and the synthetic RBS, RBS*
pEERM3-*aceA*	As pEERM3, but with *E. coli aceA* expressed from the P*nrsB* promoter
pEERM3-*aceAB*	As pEERM3, but with *E. coli aceAB* expressed from the P*nrsB* promoter

### Transformation of *Synechocystis* PCC 6803

The *Synechocystis* PCC 6803 engineered strains, containing the Km antibiotic cassette (WT + Km^r^) and overexpressing the native PEPc (WT + 2xPEPc) [37] were transformed as described previously [64]. When colonies appeared, positive transformants were identified by PCR and streaked on plates repeatedly until the constructs were fully segregated. The engineered strains created are summarized in Table 1.

### Succinate measurements

The engineered strains (WT_C, 2P_C, 2P_I and 2P_IM, see Table 1) were pre-cultivated in bubbling flasks (800 mL) containing BG11 with addition of Km (25 µg ml^−1^) and Cm (20 µg ml^−1^) under 20 µE m^−2^ s^−1^ until the OD_750_ was approximately 1 (Varian 50 Bio UV–Visible Spectrophotometer). The cells were centrifuged at 5000 rpm for 15 min at room temperature and resuspended in 30 mL of BG11 or BG11_0_ containing Km (25 µg ml^−1^) and Cm (20 µg ml^−1^). This step was repeated twice. Then, the cells were induced with 5 µM of NiCl_2_ and 1 mM of TTA was added for inhibition of SDH, when appropriate. For the condition of supplemented acetate and blocked SDH, BG11 or BG11_0_ were prepared with 50 mM of Tris pH 7.5, 0.2% Acetic acid glacial, 1 mM of TTA, Km (25 µg ml^−1^), Cm (20 µg ml^−1^) and 5 µM of NiCl_2_. 9 mL of cells (3 mL per biological replicate) were used for each condition. In light, the cells were placed in (5 mL) 6 well plates, sealed with surgical tape, under 20 µE m^−2^ s^−1^, at 30 °C under constant shaking. In darkness, the cells were placed in 6 well plates wrapped with aluminium foil, at 30 °C under constant shaking. For anoxic darkness, the cells were placed into 6 mL headspace vial, from Sigma (27293) and sealed. The vials were wrapped with aluminium foil and flushed with nitrogen or argon gas for 5 min before incubation, at 30 °C under constant shaking. After 4 days, the OD_750_ was measured using the Varian 50 Bio UV–Visible Spectrophotometer and the culture was centrifuged at 13,000 rpm for 10 min at room temperature. The supernatant was transferred to a new tube and the remaining cells were used for the Western blot analysis.

For determination of succinate levels in samples, supernatants were analysed by high pressure liquid chromatography–electrospray ionisation mass spectrometry (HPLC–ESI–MS). A solution of d4-succinate (Sigma-Aldrich USA) was prepared to 21.3 mg/L in deionised water as an internal standard. Standards were prepared in the range 0.2–40 mg/L succinate in deionised water (18.2 MΩ; MilliQ, Millipore), these were diluted with the internal standard 1:1 by pipetting 20 µL solution into 20 µL internal standard solution. Samples were diluted into the internal standard in the same way. The concentration of succinate in samples was determined by analysing the ratio of succinate: d4-succinate in the resulting solutions, and comparing with a linear calibration curve from the standards measured in triplicates.

Chromatography was performed with an Agilent 1100 HPLC using hydrophilic interaction chromatography (HILIC) with mobile phase A as acetonitrile (LichroSolv isocratic grade, Merck) and mobile phase B as 10 mM ammonium acetate (> 99%, HiperSolv Chromanorm, VWR) in deionised water (18.2MΩ; MilliQ, Millipore). The column was a Hilicon iHILIC prototype (100 × 2.1 mm, 3.5 µm particle size). Samples (0.5 µL) were injected and separated using an isocratic method at 40 °C with 70% mobile phase B. Eluting material was analysed by ESI–MS in negative mode with an Orbitrap LTQ-Velos (Thermo Fisher). The ESI–MS was operated at − 2.5 kV with sweep gas set to 2, sheath gas set to 30 and capillary temperature set to 300 °C. The Orbitrap was tuned to the signal of the deuterated standard prior to analysis and was operated at resolution setting 60,000 over mass range 115–125 Da. Succinate and d4-succinate (m/z 117.01933 and 121.04444) intensities were recorded a peak was found within 10 ppm (∆m/m) of the true deprotonated mass. Average background signal intensity from 1 to 1.5 min was subtracted and signal was summed from 1 min before to 1.5 min after the apex of the d4-succinate peak (~ 2.7 min).

### SDS-PAGE and Western immunoblot

Crude protein extraction was performed as described by [65]. The protein concentration was measured with *DC* protein assay (Bio-Rad), using albumin from bovine serum (Sigma Aldrich) as standard. For sodium dodecyl sulfate polyacrylamide gel electrophoresis (SDS-PAGE), the standard condition (200 V for 30 min) was used to run Mini-Proteian TGX Stain-free gels (any kDa) from Bio-Rad. The gels were either stained with QC Colloidal Coomassie stain (Bio-Rad) either blotted to PVDF membrane. Western immunoblot was performed according to standard protocols, 5 µg of crude extract was loaded, 5% non fat dried milk (AppliChem) was used, the rabbit anti-strep-tag II antibody (polyclonal from Abcam, 1:1000) and the goat anti-rabbit IgG (H&L) HRP conjugated from Agrisera (1:10,000) were used for detection of Strep-proteins.

For detection of PEPc, the procedure was similar with the exception that 31 µg of crude extra was loaded, 10% of ECL™ Blocking Agent from Amersham was used and the primary antibody rabbit anti-PEPc (polyclonal from Agrisera (AS09458), diluted 1:300) was incubated overnight at 4 ℃.

## Supplementary Information

**Additional file 1. **Succinate accumulated in the media (µg ml^−1^ OD^−1^) in the engineered strains (Table 1) under different conditions (light, darkness and anoxic darkness) in the presence or absence of nitrate.

**Additional file 2. **Statistical analysis showing the p values obtained when the Student's two-tailed *t*-test was performed comparing succinate titers in the media (Additional file 1) of the different strains under the same conditions.

**Additional file 3. ** Statistical analysis showing the p values obtained when the Student's two-tailed t-test was performed comparing succinate titers in the media (Additional file 1) of the same strain between B11 and BG11_0_ media under the same conditions.

**Additional file 4. **Statistical analysis showing the p values obtained when the Student's two-tailed t-test was performed comparing succinate titers in the media (Additional Table 1) of the same strain between different conditions in Light, Dark and Anoxic darkness.

**Additional file 5. **Statistical analysis showing the p values obtained when the Student's two-tailed *t*-test was performed comparing succinate titers in the media (Additional file 1) of the same strain between different light conditions but after same treatments.

## Data Availability

The datasets used and/or analysed during the current study are available from the corresponding author on reasonable request.

## Abbreviations

2-OG: 2-Oxoglutarate
2-OGDC: 2-Oxoglutarate decarboxylase
2PG: 2-Phosphoglycolate
ACO: Aconitase
Cm: Chloramphenicol
CS: Citrate synthase
FUM: Fumarase
GABA: γ-Aminobutyrate
GS-GOGAT: Glutamine synthetase-glutamate synthase pathway
ICL: Isocitrate dehydrogenase
IDH: Isocitrate dehydrogenase
Km: Kanamycin
LB: Luria-Bertani
MDH: Malate dehydrogenase
ME: Malic enzyme
MS: Malate synthase
OD: Optical density
OAA: Oxaloacetate
PDH: Pyruvate dehydrogenase complex
PEP: Phosphoenolpyruvate
PEPc: Phosphoenolpyruvate carboxylase
PEPCK: Phosphoenolpyruvate carboxykinase
PK: Pyruvate kinase
PPS: Phosphoenolpyruvate synthase
SDH: Succinate dehydrogenase
SSADH: Succinic semialdehyde dehydrogenase
SucCD: Succinyl-CoA synthase
*Synechocystis*: *Synechocystis* sp. PCC 6803
TCA: Tricarboxylic acid cycle
TTA: 2-Thenoyltrifluoroacetone
WB: Western immunoblot
