# Supplementary material for: Production of succinate by engineered strains of Synechocystis PCC 6803 overexpressing phosphoenolpyruvate carboxylase and a glyoxylate shunt
Source: Microb Cell Fact. 2021 Feb 8;20:39. doi: 10.1186/s12934-021-01529-y (PMC7871529; doi:10.1186/s12934-021-01529-y)
Supplement: Supplementary file 1 — Additional file 1. Succinate accumulated in the media (µg ml−1 OD−1) in the engineered strains (Table 1) under different conditions (light, darkness and anoxic darkness) in the presence or absence of nitrate. [file 12934_2021_1529_MOESM1_ESM.docx]

|  | **Light** | | | | | | **Dark** | | | | | | **Anoxic darkness** | | | | | |
| --- | --- | --- | --- | --- | --- | --- | --- | --- | --- | --- | --- | --- | --- | --- | --- | --- | --- | --- |
|  | **BG11** | | | **BG11_0_** | | | **BG11** | | | **BG11_0_** | | | **BG11** | | | **BG11_0_** | | |
|  | **A** | **B** | **C** | **A** | **B** | **C** | **A** | **B** | **C** | **A** | **B** | **C** | **A** | **B** | **C** | **A** | **B** | **C** |
| **WT_C** | **50.5±** 5.5 | **15.0 ±**  1.1 | **786.3**  **±**  45.4 | **33.1 ±** 6.7 | **342.4±** 47.3 | **121.0 ±** 13.1 | **109.5±** 30.9 | **681.3** **±**  118 | **440.0 ±** 88.5 | **34.8 ±** 7.0 | **557.1 ±** 53.0 | **184.7** **±** 16.0 | **179.4 ±**  100 | **128.8±** 11.1 | **158.9 ±** 19.5 | **121.9 ±** 32.5 | **114.4±** 16.3 | **168.5 ±** 44.1 |
| **2P_C** | **23.7±** 6.2 | **246.5±** 21.1 | **1616**  **±** 183 | **50.0**  **±** 8.7 | **226.6** **±** 4.3 | **407.8** **±** 15.0 | **31.1** **±** 3.7 | **783.7** **±** 98.7 | **1413**  **±** 217 | **5.4** **±** 9.3 | **646.3 ±** 44.2 | **643.6 ±** 80.0 | **678.3 ±** 191.5 | **234.5 ±** 56.2 | **1076**  **±**  119 | **282.3 ±** 30.3 | **124.2 ±** 1.4 | **527.7 ±** 66.6 |
| **2P_I** | **23.5 ±** 2.7 | **732.4 ±** 29.3 | **1157 ±**  211 | **23.2 ±** 12.4 | **440.7 ±** 9.4 | **493.6 ±** 102 | **57.7 ±** 13.8 | **2217**  **±** 37.0 | **706.5 ±** 209 | **33.3 ±** 5.3 | **1836**  **±** 115 | **578.7** **±**  190 | **1258**  **±**  472 | **255.9 ±** 9.3 | **701.2 ±** 58.9 | **301.7±** 43.9 | **220.8 ±** 115 | **622.6 ±** 56.5 |
| **2P_IM** | **74.6 ±** 10.4 | **467.2** **±** 91.4 | **1526**  **±** 84.1 | **134.1±** 17.1 | **237.7 ±** 13.4 | **692.5 ±** 105.2 | **408.1** **±** 39.9 | **986.1**  **±**  278 | **984.5 ±** 45.3 | **44.3 ±** 11.9 | **815.6 ±** 75.1 | **573.6 ±** 73.4 | **814.3 ±** 26.8 | **370.3**  **±** 208 | **700.1 ±** 72.4 | **303.4±** 16.1 | **158.2 ±** 33.6 | **560.8 ±** 30.1 |

**Additional file 1: Succinate production (µg · ml^-1^· OD^-1^) in the engineered strains (Table 1) under different conditions (light - 20 µE·m^-2^·s^-1^, darkness and anoxic darkness) in the presence or absence of nitrate.** **A** corresponds to 5 µM of NiCl_2_; **B** corresponds to 5 µM of NiCl_2_ and the addition of 2-Thenoyltrifluoroacetone (1 mM); **C** corresponds to 5 µM of NiCl_2_, the addition of 2-Thenoyltrifluoroacetone (1 mM) and 50 mM Tris pH 7.5 and 0.2% acetate. BG11 corresponds to media with the presence of nitrate; BG11_0_ corresponds to media without of nitrate. All the conditions contained Km (25 µg · mL^-1^) and Cm (20 µg · mL^-1^).
