# Supplementary material for: Production of succinate by engineered strains of Synechocystis PCC 6803 overexpressing phosphoenolpyruvate carboxylase and a glyoxylate shunt
Source: Microb Cell Fact. 2021 Feb 8;20:39. doi: 10.1186/s12934-021-01529-y (PMC7871529; doi:10.1186/s12934-021-01529-y)
Supplement: Supplementary file 2 — Additional file 2. Statistical analysis showing the p values obtained when the Student's two-tailed t-test was performed comparing succinate titers in the media (Additional file 1) of the different strains under the same conditions. [file 12934_2021_1529_MOESM2_ESM.docx]

|  | **Light** | | | | | | **Dark** | | | | | | **Anoxic darkness** | | | | | |
| --- | --- | --- | --- | --- | --- | --- | --- | --- | --- | --- | --- | --- | --- | --- | --- | --- | --- | --- |
|  | **BG11** | | | **BG11_0_** | | | **BG11** | | | **BG11_0_** | | | **BG11** | | | **BG11_0_** | | |
|  | **A** | **B** | **C** | **A** | **B** | **C** | **A** | **B** | **C** | **A** | **B** | **C** | **A** | **B** | **C** | **A** | **B** | **C** |
| **WT_C - 2P_C** | **0.047** | **0.003** | **0.023** | **0.034** | 0.059 | **<0.001** | **0.042** | 0.211 | **0.006** | 0.086 | **0.027** | **0.009** | 0.077 | 0.113 | **0.007** | **0.044** | 0.372 | 0.057 |
| **WT_C - 2P_I** | **0.028** | **<0.001** | 0.096 | 0.097 | 0.055 | **0.023** | **0.036** | **0.003** | 0.076 | 0.633 | **<0.001** | 0.080 | 0.058 | **0.017** | **0.003** | 0.053 | 0.405 | 0.075 |
| **WT_C -2P_IM** | 0.119 | **0.013** | **0.009** | **0.007** | 0.056 | **0.012** | **0.015** | 0.089 | **0.004** | 0.201 | 0.065 | **0.007** | **0.004** | 0.195 | **0.009** | **0.023** | 0.260 | 0.084 |
| **2P_C -2P_I** | 0.945 | **<0.001** | 0.062 | **0.025** | **0.001** | 0.313 | 0.055 | **0.001** | **0.015** | 0.061 | **0.001** | 0.651 | 0.271 | 0.621 | **0.033** | 0.153 | 0.445 | 0.271 |
| **2P_C -2P_IM** | **0.011** | **0.047** | 0.257 | **0.004** | 0.273 | 0.054 | **0.004** | 0.312 | 0.052 | 0.084 | 0.132 | 0.280 | 0.371 | 0.292 | **0.009** | **0.018** | 0.234 | 0.599 |
| **2P_I - 2P_IM** | **0.009** | **0.046** | 0.073 | **0.005** | **0.003** | 0.050 | **0.006** | **0.020** | 0.133 | 0.279 | **0.010** | 0.976 | 0.248 | 0.660 | 0.986 | 0.926 | 0.571 | 0.070 |

**Additional file 2: Statistical analysis showing the p values obtained when the Student's two-tailed *t*-test was performed comparing the succinate production (Additional file 1) of the different strains under the same conditions.** Light corresponds to 20 µE·m^-2^·s^-1^; BG11 corresponds to media with the presence of nitrate, BG11_0_ corresponds to media without of nitrate; **A** corresponds to 5 µM of NiCl_2_; **B** corresponds to 5 µM of NiCl_2_ and the addition of 2-Thenoyltrifluoroacetone (1 mM); **C** corresponds to 5 µM of NiCl_2_, the addition of 2-Thenoyltrifluoroacetone (1 mM) and 50 mM Tris pH 7.5 and 0.2% acetate. All the conditions contained Km (25 µg · mL^-1^) and Cm (20 µg · mL^-1^). Bold correspond to significant differences, p < 0.050.
