# Supplementary material for: Production of succinate by engineered strains of Synechocystis PCC 6803 overexpressing phosphoenolpyruvate carboxylase and a glyoxylate shunt
Source: Microb Cell Fact. 2021 Feb 8;20:39. doi: 10.1186/s12934-021-01529-y (PMC7871529; doi:10.1186/s12934-021-01529-y)
Supplement: Supplementary file 3 — Additional file 3. Statistical analysis showing the p values obtained when the Student's two-tailed t-test was performed comparing succinate titers in the media (Additional file 1) of the same strain between B11 and BG110 media under the same conditions. [file 12934_2021_1529_MOESM3_ESM.docx]

|  | **Light** | | | **Dark** | | | **Anoxic darkness** | | |
| --- | --- | --- | --- | --- | --- | --- | --- | --- | --- |
|  | **BG11 - BG11_0_** | | | **BG11 - BG11_0_** | | | **BG11 - BG11_0_** | | |
|  | **A** | **B** | **C** | **A** | **B** | **C** | **A** | **B** | **C** |
| **WT_C** | **0.002** | **0.007** | **<0.001** | 0.074 | 0.093 | 0.051 | 0.460 | 0.391 | 0.894 |
| **2P_C** | **0.048** | 0.257 | **0.008** | **0.030** | 0.176 | **0.026** | 0.084 | 0.076 | **0.017** |
| **2P_I** | 0.974 | **0.004** | 0.067 | 0.112 | **0.045** | 0.145 | 0.063 | 0.759 | **0.047** |
| **2P_IM** | 0.055 | 0.056 | **0.006** | **0.003** | 0.472 | **0.021** | **0.001** | 0.244 | 0.093 |

**Additional file 3: Statistical analysis showing the p values obtained when the Student's two-tailed t-test was performed comparing the succinate production (Additional file 1) of the same strain between B11 and BG11_0_ media under the same conditions.** Light corresponds to 20 µE·m^-2^·s^-1^; BG11 corresponds to media with the presence of nitrate, BG11_0_ corresponds to media without of nitrate; **A** corresponds to 5 µM of NiCl_2_; **B** corresponds to 5 µM of NiCl_2_ and the addition of 2-Thenoyltrifluoroacetone (1 mM); **C** corresponds to 5 µM of NiCl_2_, the addition of 2-Thenoyltrifluoroacetone (1 mM) and 50 mM Tris pH 7.5 and 0.2% acetate. All the conditions contained Kanamycin (25 µg · mL^-1^) and Chloramphenicol (20 µg · mL^-1^). Bold correspond to significant differences, p < 0.050.
