# Supplementary material for: Production of succinate by engineered strains of Synechocystis PCC 6803 overexpressing phosphoenolpyruvate carboxylase and a glyoxylate shunt
Source: Microb Cell Fact. 2021 Feb 8;20:39. doi: 10.1186/s12934-021-01529-y (PMC7871529; doi:10.1186/s12934-021-01529-y)
Supplement: Supplementary file 4 — Additional file 4. Statistical analysis showing the p values obtained when the Student's two-tailed t-test was performed comparing succinate titers in the media (Additional Table 1) of the same strain between different conditions in Light, Dark and Anoxic darkness. [file 12934_2021_1529_MOESM4_ESM.docx]

|  | **Light** | | | | **Dark** | | | | **Anoxic darkness** | | | |
| --- | --- | --- | --- | --- | --- | --- | --- | --- | --- | --- | --- | --- |
|  | **BG11** | | **BG11_0_** | | **BG11** | | **BG11_0_** | | **BG11** | | **BG11_0_** | |
|  | **A-B** | **A-C** | **A-B** | **A-C** | **A-B** | **A-C** | **A-B** | **A-C** | **A-B** | **A-C** | **A-B** | **A-C** |
| **WT_C** | **0.009** | **0.001** | **0.006** | **0.014** | **0.010** | **0.010** | **0.003** | **0.001** | 0.501 | 0.789 | 0.743 | 0.172 |
| **2P_C** | **0.003** | **0.004** | **0.001** | **0.001** | **0.005** | **0.008** | **0.002** | **0.006** | 0.090 | **0.017** | **0.013** | **0.042** |
| **2P_I** | **<0.001** | **0.012** | **0.001** | **0.019** | **<0.001** | **0.029** | **0.001** | **0.039** | 0.226 | 0.193 | 0.580 | **0.022** |
| **2P_IM** | **0.017** | **0.001** | **0.002** | **0.010** | 0.064 | **0.003** | **0.003** | **0.005** | 0.052 | 0.103 | **0.012** | **0.008** |

**Additional file 4: Statistical analysis showing the p values obtained when the Student's two-tailed t-test was performed comparing the succinate production (Additional table 1) of the same strain between different conditions in Light, Dark and Anoxic darkness.** Light corresponds to 20 µE·m^-2^·s^-1^; BG11 corresponds to media with the presence of nitrate, BG11_0_ corresponds to media without of nitrate; **A** corresponds to 5 µM of NiCl_2_; **B** corresponds to 5 µM of NiCl_2_ and the addition of 2-Thenoyltrifluoroacetone (1 mM); **C** corresponds to 5 µM of NiCl_2_, the addition of 2-Thenoyltrifluoroacetone (1 mM) and 50 mM Tris pH 7.5 and 0.2% acetate. All the conditions contained Kanamycin (25 µg · mL^-1^) and Chloramphenicol (20 µg · mL^-1^). Bold correspond to significant differences, p < 0.050.
