# Supplementary material for: Production of succinate by engineered strains of Synechocystis PCC 6803 overexpressing phosphoenolpyruvate carboxylase and a glyoxylate shunt
Source: Microb Cell Fact. 2021 Feb 8;20:39. doi: 10.1186/s12934-021-01529-y (PMC7871529; doi:10.1186/s12934-021-01529-y)
Supplement: Supplementary file 5 — Additional file 5. Statistical analysis showing the p values obtained when the Student's two-tailed t-test was performed comparing succinate titers in the media (Additional file 1) of the same strain between different light conditions but after same treatments. [file 12934_2021_1529_MOESM5_ESM.docx]

|  | **Light-Darkness** | | | | | | **Light-Anoxic darkness** | | | | | |
| --- | --- | --- | --- | --- | --- | --- | --- | --- | --- | --- | --- | --- |
|  | **BG11** | | | **BG11_0_** | | | **BG11** | | | **BG11_0_** | | |
|  | **A** | **B** | **C** | **A** | **B** | **C** | **A** | **B** | **C** | **A** | **B** | **C** |
| **WT_C** | 0.093 | **0.010** | **0.040** | 0.798 | **0.059** | **0.012** | 0.164 | **0.004** | **0.003** | 0.054 | **0.018** | 0.277 |
| **2P_C** | 0.086 | **0.012** | 0.342 | **0.017** | **0.012** | 0.342 | **0.026** | 0.762 | **0.050** | **0.004** | **0.001** | 0.081 |
| **2P_I** | **0.035** | **0.001** | 0.154 | 0.407 | **0.002** | 0.363 | **0.046** | **0.007** | 0.060 | **0.005** | 0.203 | 0.153 |
| **2P_IM** | **0.004** | 0.095 | **0.014** | **0.022** | **0.004** | 0.366 | **<0.001** | 0.303 | **0.009** | **0.008** | **0.023** | 0.119 |

**Additional file 5: Statistical analysis showing the p values obtained when the Student's two-tailed *t*-test was performed comparing the succinate production (Additional file 1) of the same strain between different light conditions but after same treatments.** Light corresponds to 20 µE·m^-2^·s^-1^; BG11 corresponds to media with the presence of nitrate, BG11_0_ corresponds to media without of nitrate; **A** corresponds to 5 µM of NiCl_2_; **B** corresponds to 5 µM of NiCl_2_ and the addition of 2-Thenoyltrifluoroacetone (1 mM); **C** corresponds to 5 µM of NiCl_2_, the addition of 2-Thenoyltrifluoroacetone (1 mM) and 50 mM Tris pH 7.5 and 0.2% acetate. All the conditions contained Kanamycin (25 µg · mL^-1^) and Chloramphenicol (20 µg · mL^-1^). Bold correspond to significant differences, p < 0.050.
